# Implementing Silica Nanoparticles in the Study of the Airborne Transmission of SARS-CoV-2

**DOI:** 10.3390/molecules27123896

**Published:** 2022-06-17

**Authors:** Robert Hildebrandt, Krystian Skubacz, Izabela Chmielewska, Zdzisław Dyduch, Aleksandra Zgórska, Adam Smoliński

**Affiliations:** 1 Department of Underground Research and Surface Maintenance, Central Mining Institute, Podleska 72, 43-190 Mikołów, Poland; 2 Silesian Centre for Environmental Radioactivity, Central Mining Institute, Plac Gwarków 1, 40-166 Katowice, Poland; kskubacz@gig.eu (K.S.); ichmielewska@gig.eu (I.C.); 3 Department of Dust Hazard Control, Central Mining Institute, Podleska 72, 43-190 Mikołów, Poland; zdyduch@gig.eu; 4 Department of Water Protection, Central Mining Institute, Plac Gwarków 1, 40-166 Katowice, Poland; azgorska@gig.eu; 5 Central Mining Institute, Plac Gwarków 1, 40-166 Katowice, Poland

**Keywords:** SARS-CoV-2, bioaerosol, airborne transmission, SEM, TEM, silica nanoparticle marker

## Abstract

Aerosol transmission constitutes one of the major transmission routes of the SARS-CoV-2 pathogen. Due to the pathogen’s properties, research on its airborne transmission has some limitations. This paper focuses on silica nanoparticles (SiO_2_) of 40 and 200 nm sizes as the physicochemical markers of a single SARS-CoV-2 particle enabling experiments on the transmission of bioaerosols in public spaces. Mixtures of a determined silica concentration were sprayed on as an aerosol, whose particles, sedimented on dedicated matrices, were examined by scanning electron microscopy (SEM) and transmission electron microscopy (TEM). Since it was not possible to quantitatively identify the markers based on the obtained images, the filters exposed with the AirSampler aspirator were analyzed based on inductively coupled plasma optical emission spectroscopy (ICP-OES). The ICP-OES method enabled us to determine the concentration of silica after extracting the marker from the filter, and consequently to estimate the number of markers. The developed procedure opens up the possibility of the quantitative estimation of the spread of the coronavirus, for example in studies on the aerosol transmission of the pathogen in an open environment where biological markers—surrogates included—cannot be used.

## 1. Introduction

The global death toll caused by coronavirus SARS-CoV-2 has now topped 5.3 million [1,2,3,4,5,6]. There are three main models of respiratory virus transmission: (1) contact, when the transmission happens through direct contact with infected people (e.g., hand shaking) or surfaces; (2) droplets, when infection occurs via the direct deposition of virus-containing large respiratory droplets (>5 μm) of the mouth, nose, or eyes; and (3) aerosol transmission, during which infection occurs by inhaling small droplets (<5 μm). The first two models involve so-called non-airborne transmission, while the third model involves airborne transmission [7]. The aerosol transmission model constitutes one of the most dominant routes of the SARS-CoV-2 pathogen’s spread [8,9,10,11]. Liu et al. [3] and Zhou et al. [12] confirmed the spread of the SARS-CoV-2 pathogen through aerosol transmission in hospital conditions, which led to the assumption that aerosols also take part in the pathogen’s spread in public spaces. The course of coronavirus transmission may be described through the relationship characterizing the spread of aerosols, the initial size distribution of the complexes, and the concentration and size of the aerosols present in the environment, as well as environmental conditions such as humidity, temperature, and pressure [13]. Additionally, the behavior of virus particles in an aerosol or bioaerosol is also determined by its structure and morphology, including the presence of a large amount of viral and cellular proteins and cellular debris, which significantly changes the rate of evaporation of liquid from the particles, and consequently determines the residence time of the virus particles in the aerosol. The above-mentioned factors have an impact on the size of the exhaled complexes. Larger objects with sizes exceeding 100–200 µm do not persist in the air and fall under gravity. The behavior of smaller particles is similar to that of aerosols; depending on the ventilation conditions, they may travel larger distances, and consequently may enter the organisms of other people. Higher temperatures favor evaporation and a decrease in aerosol size, whereas higher humidity is conducive to increasing the size. In turn, higher concentrations of environmental aerosols result in increased coagulation efficiency. Skubacz et al. [14] in their studies conducted under the conditions of forced ventilation proved that the discussed phenomena take place within a fraction of a second. Hinds [13] described the initial mechanisms concerning the formation and transmission of aerosols; however, due to the complexity of the processes, their modeling and analytical assessment pose a considerable challenge. The application of markers that imitate viruses and the simulation of their transmission routes in the environment constitute attempts to address the problem.

The increasing virological risks determine the need for research in the area of pathogen identification and transmission pathways. Due to the complexity of the problem, these issues have not been sufficiently explored so far. The quantitative and qualitative identification of microorganisms present in bioaerosols is based mainly on conducting growth method analyses, as well as molecular and immunological tests, and mainly concerns the detection of pathogens such as bacteria, fungi, or allergens [15]. Very few studies deal with the quantitative and qualitative identification of viruses occurring in aerosols [16,17]. This is mainly due to the properties of pathogens [18,19]. The fact that the coronavirus belongs to the 3rd Risk Group of Viruses and Prions [20] makes it necessary to conduct research work according to adequate safety standards [21]. The high pandemic potential of SARS-CoV-2 determines the necessity of using microbial surrogates or physicochemical markers of a similar size to the coronavirus during research, which will be easy to identify, harmless to personnel, and allow the use of developed mathematical formulas.

The surrogates constitute the most proximate taxonomic group of microorganisms characterized by significantly lower or insignificant virulence and harmfulness, and their use allows for recognizing the mechanisms of the examined processes [22,23]. The literature describes the use of HCoV-229E and OC-45 strains as surrogates of SARS-CoV-2 [22,24]. Also noteworthy is the work describing aerosol tracer (DNA-tagged tracers) testing in airplane conditions. The authors conducted a simulation study of the spread of a marker in air using fluorescent and DNA-tagged microspheres. During the research, fluorescent 1 μm and DNA-tagged 3 μm particles were released and measured in multiple rows and seats distributed throughout each aircraft and the research results provided a basis for evaluating the pathway of the viral spread [25].

The physical (abiotic) markers constitute an alternative to the microbiological surrogates. This group includes nanoparticles and fluorescent markers (fluorochromes, Ø2–10 nm) used in cytometric analyses [26]. To date, several models of flow cytometers for detecting viruses of the 100 to 900 nm size range have been developed; nevertheless, the systems are dedicated to the identification of pathogens in liquid samples and they have not found an application in bioaerosols [27].

Within the group of abiotic markers, the silica markers deserve special attention due to their size being equal to coronavirus particles, their low cost, the simplicity of their application and identification (analytical and microscopic techniques), and their neutral character, allowing their use in real conditions in non-isolated environments [28,29]. In the field literature, numerous techniques for silica measurements are reported, depending on the analyzed matrix.

The US National Institute for Occupational Safety and Health (NIOSH) approved a few methods for the detection of crystalline silica. In the NIOSH 7500 method [30], dust containing silica is collected on polyvinyl chloride (PVC) and is ashed in a muffle furnace for 2 h at the temperature of 600 °C. The dry residue with 2-propanol is redeposited on a silver filter and analyzed utilizing XRD (X-ray diffraction) [31]. The NIOSH Method 7602 [32] involves the collection of airborne silica on a PVC filter, along with filter ashing and pellet preparation with KBr. Finally, the samples are detected using Fourier-transform infrared spectroscopy (FTIR). IR method is believed to be accurate for small quantities of crystalline silica; however, the other components of the matrix may interfere and limit the detection threshold [33]. The NIOSH 7601 method is used less extensively [34]. Air dust is collected on a mixed cellulose ester membrane and silica is digested in nitric acid (HNO_3_), phosphoric acid (H_3_PO_4_), and hydrofluoric acid (HF). Finally, the analyte in the form of silicon complexes (silico-molybdate and molybdenum blue) is measured through visible adsorption spectrophotometry. Methods employing a filter-based collection of airborne silica have a few drawbacks, which include the reasonably high detection limits (ranging from 1 up to 10 µg per sample) and the long sample collection time. 

The measurement of silica aerosols employing quantum cascade laser (QCL)-based infrared spectroscopy seems to be a more promising option taking into account the detection limit and sample collection time. Silica-containing particulate matter is deposited on a filter membrane and then undergoes detection in a system as described by Wei et al. [35]. Wei et al. [35] tested a few types of filters and different methods for silica aerosol deposition and found that polyvinyl chloride and polycarbonate filters have a better signal-to-noise ratio compared to polypropylene filters. The detection limit of this technique allows one to attain about 3.3 µg/sample within a relatively short measurement time.

Silicon occurring in nature has three stable isotopes, 28Si, 29Si, and 30Si, with percentage abundances of 92.2%, 4.7%, and 3%, respectively. This phenomenon facilitates silica determination through HR-ICP-MS (high-resolution inductively coupled plasma mass spectrometry). Yu et al. [36] firstly deposited crystalline quartz on the PVC filters. In the next stage, each filter was digested in HF, the obtained solution was presented to a nebulizer, and the generated aerosols were measured using a high-resolution mass spectrometer. The detection limit for this method is 8 ng Si/g. 

The possibility of identifying the markers on filter materials opens up numerous research paths within the scope of virus transport associated with aerosols. Markers sprayed in the air are collected on the device’s filters. Such devices are equipped with separation systems, which enable the separation of the so-called aerosol respirable fraction, the sizes of which do not exceed 10 µm and which according to the ISO [37] criteria can penetrate the non-ciliated region of the respiratory tract.

In the case of “active” aerosols, also the thoracic fraction is capable of passing beyond the larynx as well as the respirable class, including all kinds of suspended dust inhaled through the nose and mouth, which may result in an infection. As for the transport of the aerosols, this depends to a large extent on their size, which necessitates the application of devices enabling one to explore the size distribution of “active” aerosols, i.e., the impactors. The impactors are devices equipped with impaction plates on which aerosols of particular sizes are collected during the measurements of diffusion screens, which capture fine aerosols [13,38].

The objective of the paper was to develop a method that could be used for the investigation of the transmission of the SARS-CoV-2 pathogen based on the application of an abiotic SiO_2_ marker. The research was conducted using the developed proprietary procedure for the assessment of the SARS-CoV-2 pathogen transmission, the aim of which was to quantitatively estimate the spread of the coronavirus markers. This study was supposed to allow a better understanding of the mechanisms of the pathogen spread in the environment in situations when biological markers, including surrogates, cannot be used. However, it should be emphasized that at this stage the aim of the article was not to model phenomena but just to develop a method to identify markers on a filter. Modeling the aerosol spread will be the next step by using devices that can separate aerosols into different size classes. Aerosol transmission constitutes one of the most dominant routes of the SARS-CoV-2 pathogen’s spread. Therefore, this work was concentrated on air-borne SARS-CoV-2. This was because the authors intended to perform such experiments in an underground environment with forced ventilation, where viruses can travel a long distance from the source to the receivers along the air stream.

## 2. Materials and Methods

For the purpose of this research, solutions with silica markers measuring 40 and 200 nm were prepared and next sprayed in the form of aerosols on dedicated matrices. An analysis of the collected material was performed in order to quantitatively identify the markers. The tests were made by employing scanning electron microscopes (SEM) as well as transmission electron microscopes (TEM). Several attempts at quantitatively identifying particular marker particles using the SEM technique did not bring about the expected results. Only the cryogenic fixation of the sample and a subsequent TEM observation enabled us to obtain clear images of the markers. However, while applying the TEM technique, it is difficult to precisely determine the amount of analyzed sample, which excludes the possibility of performing a quantitative analysis of the markers. Considering this limitation, for the experiments an AirSampler-type aspirator enabling the collection of the aerosols on filters was used. In the next step, the collected particles were extracted from the filters and the chemical concentration of the silica was evaluated through inductively coupled plasma optical emission spectroscopy (ICP-OES). 

The obtained results of the quantitative silica measurements on the filters from the subsequent consecutive stages of extraction were next applied to determine the efficiency of the process for both marker sizes, which in turn allowed us to estimate their numbers. The presence of the markers in the solutions after the extraction was also confirmed through TEM observations.

### 2.1. Markers and the Preparation of the Solutions

The coronavirus marker was used in the form of solid spherical silica nanoparticles suspended in water (General Engineering and Research, San Diego, CA, USA). The silica nanoparticles were synthesized using the Stöber method [39] to guarantee very high purity (+99.999%) with a narrow size distribution. Two sizes of initial silica nanoparticle solutions were used in the research, i.e., 40 nm and 200 nm. Their properties are described below in Table 1. The marker density of 2.65 g/cm^3^ is more than 1 g/cm^3^. However, the terminal settling velocity for the 40 nm particles is ca. 2 × 10—7 m/s, and for the 200 nm particles ca. 4 × 10—6 m/s, even if the Cunnigham correction factor is taken into account. The terminal velocity is reached by the particles within a timeframe of far less than 0.01 ms, so across almost the whole distance, from the height of 1.5 m to the ground, they will travel at such a velocity. This means that the retention time will be more than 2000 h for 40 nm particles and more than 100 h for 200 nm particles.

For the experimental studies, 100 mL of working solution with each silica nanoparticle size was prepared by dilution. During the dilution, 4 mL of initial silica marker solution was transferred into volumetric flasks and filled up to a 100 mL total volume with 1% ethanol. The working solutions were named R1 and R2 for 40 nm and 200 nm, respectively. The marker concentration in solution R1 was 5602 mg/L, while in R2 it was 4332 mg/L.

### 2.2. Spraying of the Solution and Aerosol Sampling

The spraying of the solutions in the form of an aerosol was performed in two series. In series I, the solution labeled R1 containing markers measuring 40 nm was used, whereas in series II, the solution labeled R2 containing markers measuring 200 nm was used. For each series, three independent experiments were conducted in a way that ensured similar experimental conditions regarding the amount of sprayed solution, the operation time of the nozzle, as well as the parameters characterizing the environment in the location of the research (Table 2).

**Figure 1:**
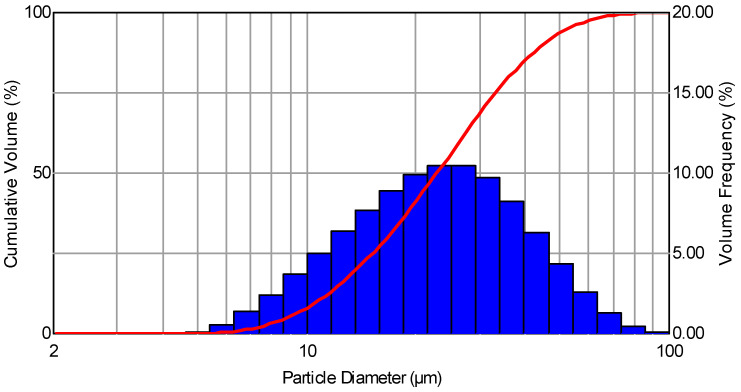
The distribution of droplet sizes generated by the Lumina ST-6R1.3 nozzle.

The aerosol was generated by employing a Lumina ST-6R1.3 nozzle (Lumina, Dongguan, China) with a diameter of 1.3 mm that met the regulation efficiency in the range of 0–360 mL/min, which was from producing a stream in the shape of a 95 mm diameter cone at a distance of 300 mm from the nozzle. The liquid was fed to the nozzle under the impact pressure of 1 m and sprayed with compressed air under the pressure of 100 kPa. The distribution of the droplet sizes of the generated aerosol is presented in Figure 1. The standardized mean difference (SMD) for the generated droplets was 19 μm.

The measurement of the droplet sizes was performed using a Spraytec (Malvern Panalytical Ltd., Malvern, UK) analyzer, which uses laser light diffraction as well as a diffraction model based on Mie and Fraunhofer scattering, including a patented multiple scattering correction method utilizing a HeNe laser light of 632.8 nm wavelength. The size of the droplets was measured within the range of 0.1–2000 μm. The tests were performed in a laboratory room of 117 m^3^ cubature under conditions of comparable temperature and humidity.

**Figure 2:**
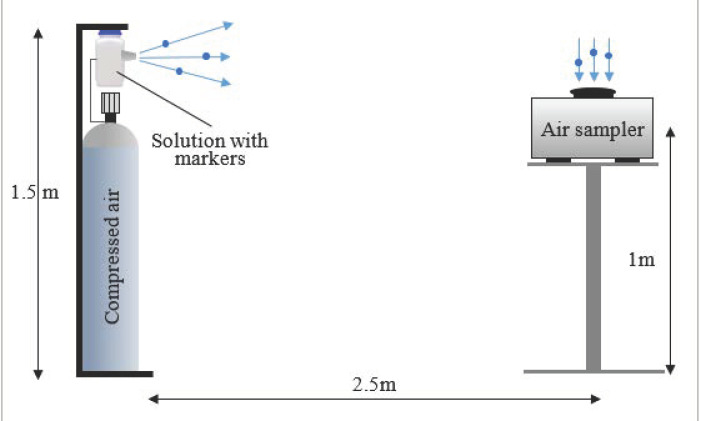
The scheme of the research stand used for examining the dispersion of the virus markers.

The stream of the generated aerosols was directed towards the SASS 3100 AirSampler (Research International Inc., Monroe, WA, USA) aspirator, which was located at a distance of 2.5 m from the aerosol nozzle and at a height of 1 m. The aspirator enabled the suction of the air including the aerosol with the determined efficiency of 200 L/min for collection on the replaceable filter. The distance between the aspirator and the aerosol nozzle was determined based on the so-called “safe social distance” of 2 m recommended by the WHO, which was enlarged by an additional safety margin of 0.5 m [40]. The scheme of the research stand is presented in Figure 2. The parameters of the filters used for the air sampling are compiled in Table 3.

The generated aerosols correspond to the size of the particles that are produced when coughing or urinating.

### 2.3. Filter Extraction Procedure and Silica Concentration Determination

The collected silica nanoparticles were extracted from the above-described filters with a manual procedure. Firstly, the filters were removed from the plastic holder and placed in 150 mL glass beakers. To each beaker, 5 mL of 1% ethanol was added, then the filters were shaken 3 times using a Vortex-Genie 2 Digital (Scientific Industries, Bohemia, NY, USA) device for 30 s at a speed of 2500 rpm. After shaking, the filters were left overnight to dry. Silica marker extraction from every filter was performed for four days running, so finally 24 liquid extracts with different silica concentrations were obtained. These were marked as R1a ex1, R1a ex2, R1a ex3, R1a ex4, R1b ex1, etc., as shown in Table 4. In the next step, the silicon content in liquid extracts was determined. For this purpose, the extracts were diluted using high-purity deionized water (conductivity below 0.05 µS/cm, Direct-Q3 UV, Millipore) and an internal standard certified silicon solution was added (AccuStandard, New Haven, CT, USA). The silicon was measured in the form of SiO_32_- using inductively coupled plasma optical emission spectrometry with the application of an Optima 5300 DV spectrometer (Perkin Elmer, Waltham, MA, USA) according to ISO standard 11885:2009 [41].

### 2.4. Imaging by Means of Electron Microscopy

#### 2.4.1. Scanning Electron Microscopy (SEM)

The imaging of silica marker in solutions sprayed on dedicated carriers was performed via scanning electron microscopy (SEM) with energy-dispersive spectroscopy (EDS) detection and a large-field detector (LFD). The SEM imaging supported with an X-ray EDS microanalysis was performed using an SEM SU3500 Hitachi electron microscope (Hitachi High-Tech Corporation, Tokyo, Japan) with a variable vacuum coordinated with an EDS UltraDry X-ray spectrometer with energy dispersion. The X-ray microanalysis was performed under the following parameters: accelerating voltage—15 keV; working distance (WD)—10 mm; pressure—30 Pa; vacuum—variable. The SEM analysis with the LFD detector and secondary electron (SE) emission was performed with the application of an ESEM Quanta 250 FEG–FEI microscope (Thermo Fisher Scientific, Waltham, MA, USA). The imaging was performed in low-vacuum (LV) mode.

#### 2.4.2. Transmission Electron Microscopy (TEM)

The TEM imaging of the silica marker was performed in R1 and R2 solutions before spraying them, as well as in solutions obtained after the fourth step of filter extraction (R1a-c ex4 and R2a-c ex4, respectively). The analyzed material was prepared via the cryogenic fixation of the tested samples (cryo-TEM). For the purpose of the research, a Tecnai F20 X-TWIN microscope (FEI Company, Hillsboro, OR, USA) was used. The microscope was equipped with a field emission gun operated at an acceleration voltage of 200 kV. The images were recorded on a Gatan Rio 16 CMOS 4k camera and processed with Gatan Microscopy Suite (GMS) software (Gatan Inc., Pleasanton, CA, USA). Specimen preparation was performed via vitrification of the aqueous solutions on grids with a holey carbon film. Before use, the grids were activated for 15 s in oxygen plasma using a Femto plasma cleaner. Cryo-samples were prepared by applying a droplet (about 3 μL) of the suspension to the grid, blotting with filter paper, and immediately freezing in liquid ethane using a fully automated blotting device. After preparation, the vitrified specimens were kept under liquid nitrogen until they were inserted into a cryo-TEM and analyzed in the TEM at −178 °C. 

## 3. Results and Discussion

**Figure 3:**
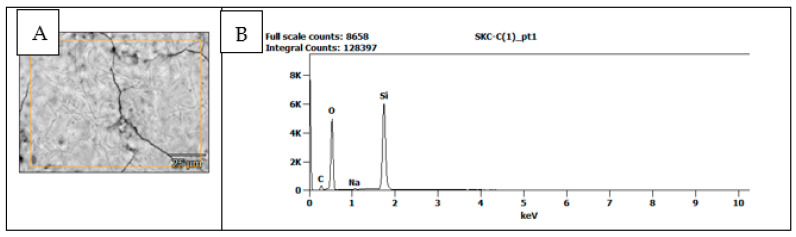
SEM image of the morphology of the aerosol droplet microarea for R1 solution (**A**) and the spectrum of the chemical content of the examined microarea (**B**).

The nozzle used in the course of the experiments enabled us to obtain aerosol droplets with sizes within the range of 2–100 µm. Considering the size distribution of the generated droplets, it was stated that 10–20 µm particles dominated in the sprayed aerosol. In the total volume of the aerosol, more than 50% of the generated droplets had a diameter below 20 µm, whereas over 90% of the generated particles had a diameter that did not exceed 50 µm. The distribution of the generated aerosols was similar to the actual droplet size distribution produced during speaking or coughing. Coughing generates on average 3000 droplets, which roughly corresponds to the number of droplets exhaled during a five-minute speech [42]. The average size of the droplets exhaled by a healthy person ranges from 0.1 to 10 μm [43]. The dominant majority of the respiratory droplet sizes of a healthy person are within the range of 0.1–8 μm, while in the case of patients, the range is 0.05–10 μm. Chao et al. [15] found that 62% of the droplets that are present in exhaled air during speaking have a diameter of <12 μm. Likewise, in the case of coughing, droplets measuring <12 μm in diameter constitute 72% of all exhaled complexes. The aerosolization that was performed in the experimental research not only facilitated the dispersion of the silica marker constituting the substitution of the human coronavirus SARS-CoV-2 but also allowed us to obtain an aerosol in which the droplet size distribution approximated the actual conditions of coronavirus transmission in public spaces, which takes place during coughing, sneezing, and speaking with COVID-19-infected individuals. In the initial phase of the research, an attempt was made to quantitatively identify the silica marker using scanning electron microscopy (SEM). Although present in the droplets of the sprayed R1 and R2 solutions, the 40 and 200 nm markers collected on the matrices dedicated to SEM tests could not be identified in the obtained SEM images. In addition, no satisfying results were achieved, even in the attempt to obtain the marker images by examining the solutions themselves before they were sprayed. The only evidence of the marker’s presence, both before and after spraying and collecting them on the matrices, was the result of the EDS detector X-ray microanalysis of the particles’ chemical content performed to determine the dominant forms of particular elements. Figure 3 presents the results of the SEM morphological analysis of the aerosol droplet microarea for the R1 solution coupled with the spectrum of the chemical content of the examined microarea. Within the scope of the research, for both the R1 and R2 solutions, a series of microanalyses before and after spraying were performed. They comprised several dozen measurements of the particles to determine the chemical forms of particular elements. The large number of attempts performed independently in several laboratories at imaging the marker, along with the similar results both in terms of the morphology and the chemical content of the microarea, suggest that the SEM/EDS technique under the conditions of the conducted experiments enables one to qualitatively identify the used markers by confirming the presence of the silica on the sprayed matrix without the possibility of quantitatively estimating the marker. The difficulty in the quantitative identification of the marker using the above technique may result from the phenomenon of solution coalescence of the silica particles in the observed area (Figure 3A) [44,45].

**Figure 4:**
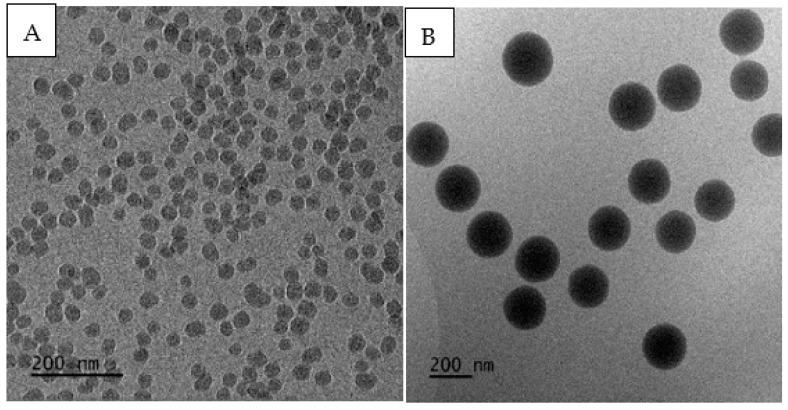
Examples of TEM images with visible silica markers: solution R1 (**A**); solution R2 (**B**).

Better imaging results, which enabled a clear distinction of single particles of the marker in the observed microarea and allowed us to determine their sizes, were achieved by employing transmission electron microscopy (TEM) with a cryo-TEM mode facilitating the physical fixation of the sample through freezing. The tests were carried out separately for each of the solutions prepared for spraying. The images of R1 and R2 solutions before spraying, as presented in Figure 4, illustrate the obtained results. Because of the adopted methodology and the objective of the research, the analyses of the microscope images after dispersing the solutions in the form of an aerosol were of key importance, which disqualified the direct use of cryo-TEM. The only solution to the problem was transforming the aerosols collected on the carriers back into a liquid, freezing the samples, and then imaging the particular markers.

**Figure 5:**
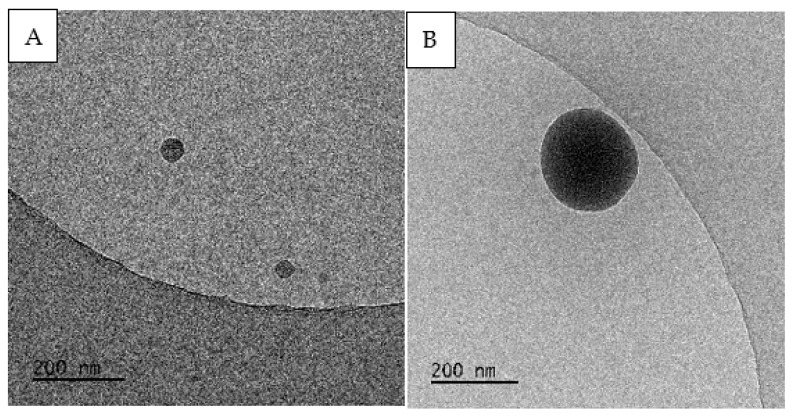
Examples of TEM images of samples after the fourth step of extraction (R1 ex4 and R2 ex4), presenting 40 nm (**A**) and 200 nm (**B**) markers, respectively.

This was made possible by the extraction of the AirSampler filters, which absorbed the sprayed aerosol droplets together with the markers. The liquid material obtained from the last steps of the filter extraction process and examined using the cryo-TEM technique contained the markers; however, their identification was problematic due to the random character of their occurrence in the observed microarea and their relatively small number concerning the volume of the tested sample (approx. 3 µL) (Figure 5).

The images obtained in this way could not provide the basis for the quantitative determination of the number of markers captured by the AirSampler aspirator filters. In addition, the mode of preparation constituted yet another obstacle because it excluded the possibility of determining the volume of the examined sample, and consequently the accurate number of markers in a given volume.

The application of the cryo-TEM technique enabled the confirmation of the presence of the silica marker in the solution after the extraction had been performed (Figure 5). Comparing the TEM images of the solution samples before (Figure 4) generating the aerosols and the solutions obtained after filter extraction (Figure 5), it was possible to observe a high concentration of the marker in solutions R1 and R2 and a very low concentration of the marker in the solutions after the last step of filter extraction. This indicates both the marker’s dispersion in the space between the nozzle and the aspirator and the good efficiency of the marker extraction process. It is significant that the application of the TEM technique facilitated the qualitative identification of the silica marker in the solutions after the extraction using the filters from the AirSampler aspirator placed at a distance of 2.5 m from the nozzle. This confirms the fact that the filters captured the aerosols containing 40 and 200 nm markers, which simulated in the research single virions of the coronavirus (SARS-CoV-2). The filters with the marker particles sedimented after spraying were subjected to a four-step extraction process using 5 mL of 1% ethanol solution. Each time, the concentration of the marker was checked in the form of SiO_3_^2−^ in the obtained extract using inductively coupled plasma optical emission spectrometry (ICP-OES).

Assuming that each subsequent step of the filter extraction runs with the same efficiency η, the mass of the silica in the solution should be:

VC_1_ = ηM_o_ (1)

VC_2_ = η(1 − η)M_o_ (2)

VC_3_ = η(1 − η)^2^M_o_ (3)

VC_4_ = η(1 − η)^3^M_o_ (4)

after the first, second, third, and fourth steps, respectively, where V = 5 mL is the volume of the solution, C_i_ is the mass concentration of silica in the solution determined in consecutive measurements using ICP-OES, and M_o_ is the unknown initial mass of silica that was sedimented on the filter during the operation of the aspirator.

As a result of applying the equations, it is possible to calculate the efficiency η, where the subscripts i and j indicate the subsequent steps of the marker extraction from the filters:

(5) $$\;\frac{{\mathrm{VC}}_{i}}{{\mathrm{VC}}_{j}}=\frac{{\eta \left(1-\eta \right)}^{i-1}M_{o}}{{\eta \left(1-\eta \right)}^{j-1}M_{o}}\;\;\;\overset{}{\Rightarrow }\eta =1-{\left(\frac{C_{i}}{C_{j}}\right)}^{\frac{1}{i-j}},\;\;i\neq j\;\;$$

In a blank sample prepared by means of rinsing a clean filter, the silica concentration in the obtained solution equaled 0.419 mg/L, which was adopted as a reference level. Table 5 presents the results of the assessment in terms of the extraction efficiency of the 40 nm markers captured by the filter, the estimated values of the initial masses of the markers M_o_ immediately after the air had been pumped through the filter, as well as their number, taking into consideration the density, size, and spherical shape, calculated as follows:

(6) $$M_{o}=\frac{VC_{i}}{{\eta \left(1-\eta \right)}^{i-1}}$$

To estimate the values of the initial masses of the markers Mo, four extraction steps were performed within the framework of three independent experiments (see Table 6, series I). It follows from the estimated values of the initial masses that the relative differences in the numbers of markers collected in the course of the research were in the range of 1–4. The efficiency was calculated by taking into account all of the extraction steps and the differences in the marker concentrations among the subsequent steps. The average efficiency of the marker extraction process based on the above mentioned experimental results was 0.558.

Taking into account only the results of the measurements of the marker concentrations after the first and fourth extraction steps and using Equation (5), efficiency values equaling 0.60, 0.53, and 0.61 were obtained. The values did not differ much from the ones presented in Table 6 for the particular experiments. To a large degree, this confirms the assumption that each subsequent step of the marker extraction process from the filters ran with a similar efficiency. Table 7; Table 8 present the results of the assessment of the extraction efficiency of the 200 nm markers captured by the filter. Four extraction steps of SiO_2_ from the filters were performed for three independent experiments (see Table 5, series II). It follows from the estimated values of the initial masses that the numbers of markers collected during the experiments were very close. Table 8 compiles the results of the calculations; only the results of the measurements of marker concentrations after the first and fourth extraction steps were taken into consideration. Regarding the obtained results, the average estimated extraction efficiency values were 0.54 (see Table 7) and 0.57 (see Table 8). Therefore, the average extraction efficiencies did not differ significantly from each other, nor were they much different from the average extraction efficiency estimated for the 40 nm markers, which was 0.56 (Table 6).

The numbers of markers collected on filters were estimated with the assumption that the efficiency of SiO_2_ sedimentation on the filters equaled 100%. Therefore, the estimated numbers of the markers on the filters did not take into account the actual filtration efficiency. The one-point characteristic of the collection efficiency indicated by the manufacturer is 50% at 0.5 µm particle diameter. Based on the above, it was stated that the actual number of markers that reach the filter could be approximately twice as much as the values provided in Table 6, Table 7 and Table 8. However, in this research, the sizes of the aerosols that reached the aspirator were not known because the size distribution of the generated aerosols was subject to transformation before reaching the filter. In the case of experiments carried out in an open environment, the actual efficiency of the filtration process can be estimated in the place where the aspirator is located by measuring the number of markers on the two filters positioned one after another.

## 4. Conclusions

The application of silica markers enables researchers to design and conduct experimental studies on the transmission of coronavirus (SARS-CoV-2) without the risk of a negative impact of the work on the research staff. Sometimes scientists use biological markers that can be used at the defined rigors. Most often, however, markers of this type have sizes that differ from the size of the coronavirus. For example, in the work by Kinahan et al. [25], the test sizes were chosen based on the existing understanding of sizes most likely to contain the SARS-CoV-2 virus, which was the area of the particle size limit within which the virus was identified (submicron (0.25 to 1 μm) and supermicron (>2.5 μm) ranges). In this publication, marker particles of a size corresponding to coronavirus (40, 200 nm) were sprayed and additionally a nozzle that generates aerosols with particle sizes equivalent to respiratory droplets generated during coughing was used.

It was confirmed that silica nanoparticles can be used as physical surrogates of the SARS-CoV-2 coronavirus on a microscale under laboratory conditions, as well as in real conditions, which constitutes a considerable advantage over the other markers and surrogates accessible on the market.

In light of the ever-emerging new strains of bacteria and viruses, using silica markers to simulate the spread of dangerous agents through airborne aerosols may prove a useful research approach to assess the transmission potential of such agents and to determine the methods of minimizing the risk of infection. The results of the SEM and TEM imaging enable us to confirm the presence of silica markers in the examined samples; however, they do not allow us to determine the number of markers in the samples because it is problematic to estimate the amount of the solution sample designated for such an analysis.

The quantitative estimation of the marker number may be achieved through the assumptions proposed in this paper and the adopted proprietary method consisting of a multi-step filter extraction process and the ICP-OES determination of silica concentrations and relevant calculations.

The results of the assessment of the efficiency of the marker extraction from filters at the level of 50–60% confirmed that the developed proprietary method, based on the ICP-OES analysis, enables researchers to analyze the spread of the coronaviruses forming complexes with environmental aerosols with the use of safe and inexpensive markers.

## Figures and Tables

**Table 1:** Properties of the liquid silica marker used in the research.

Parameter	Description	
Formula	SiO_2_ dispersed in H_2_O	
Components	Water	>80.0%
	colloidal silica	<15.0%
Appearance	form:	liquid
	colour:	clear to white
pH	7.0–8.0	
Relative density	1.00–1.20	
SiO_2_ density	2.65 g/cm^3^	

**Table 2:** The conditions of the experiment.

Series	Solution	Amount of the Solution	Time of the Nozzle Operation	Temperature [^°^C]	Humidity [%]	Sample Label (Filter)
I	R1	20 mL	7 min 18 s	19	40	R1a
	R1	20 mL	8 min 25 s	19	37	R1b
	R1	20 mL	7 min 41 s	18	38	R1c
II	R2	20 mL	8 min 04 s	19	36	R2a
	R2	20 mL	7 min 29 s	18	37	R2b
	R2	20 mL	7 min 44 s	18	37	R2c

**Table 3:** AirSampler filter parameters.

Parameter	Description
Filter Media Size	4.4 cm active diameter filter, mounted in a 6.0 cm diameter injection-molded holder
Filter Mass and Composition	12 mg/cm^3^, composed of polypropylene electret microfiber
Filter Collection Mechanism	filter discs have an electric field frozen into fibers, inducing a charge when passing through aerosols and providing effective capture
Filter Collection Efficiency	50% at 0.5 micron particle diameter

**Table 4:** Identification of solutions obtained after the extraction of silica from the filters.

Spray Phase		Extraction Phase	
Series	Solution	Filter Sample	Samples after the Extraction
I	R1	R1a	R1a ex1
			R1a ex2
			R1a ex3
			R1a ex4
		R1b	R1b ex1
			R1b ex2
			R1b ex3
			R1b ex4
		R1c	R1c ex1
			R1c ex2
			R1c ex3
			R1c ex4
II	R2	R2a	R2a ex1
			R2a ex2
			R2a ex3
			R2a ex4
		R2b	R2b ex1
			R2b ex2
			R2b ex3
			R2b ex4
		R2c	R2c ex1
			R2c ex2
			R2c ex3
			R2c ex4

**Table 5:** Marker concentrations in the solutions after extraction using 5 mL of 1% ethanol solution.

Spraying Phase				Extraction Phase	
**Series**	Solution	Filter Sample	SiO_3_^2−^ Concentration in the Solution [mg/L]	Samples after Extraction	SiO_3_^2−^ Concentration in the Solution after Extraction [mg/L]
I	R1	R1a	5602	R1a ex1	11.33
				R1a ex2	3.50
				R1a ex3	1.95
				R1a ex4	1.11
		R1b		R1b ex1	38.71
				R1b ex2	13.17
				R1b ex3	6.96
				R1b ex4	4.46
		R1c		R1c ex1	26.03
				R1c ex2	7.68
				R1c ex3	2.72
				R1c ex4	1.99
II	R2	R2a	4332	R2a ex1	23.90
				R2a ex2	16.18
				R2a ex3	4.09
				R2a ex4	2.15
		R2b		R2b ex1	25.98
				R2b ex2	8.96
				R2b ex3	5.77
				R2b ex4	2.06
		R2c		R2c ex1	22.76
				R2c ex2	8.21
				R2c ex3	5.33
				R2c ex4	2.87

**Table 6:** Extraction efficiency of 40 nm markers taking into account the results of all subsequent steps.

Extraction Efficiency of Markers from Filter *η*			Initial Mass of Markers on the Filter	Initial Number of Markers on the Filter
Average	Standard deviation	Error of the mean	M_o_	n_o_
-	-	-	(mg)	-
0.59	0.11	0.07	0.09	1.04 × 10^12^
0.51	0.14	0.08	0.37	4.21 × 10^12^
0.57	0.22	0.13	0.22	2.52 × 10^12^
0.56	0.04	0.02		

**Table 7:** Extraction efficiency of 200 nm markers taking into account the results of all subsequent extraction steps.

Extraction Efficiency of Markers from Filter *η*			Initial Mass of Markers on the Filter	Initial Number of Markers on the Filter
Average	Standard deviation	Error of the mean	M_o_	n_o_
-	-	-	(mg)	-
0.54	0.22	0.13	0.217	1.95 × 10^10^
0.58	0.18	0.10	0.221	1.99 × 10^10^
0.51	0.14	0.08	0.220	1.98 × 10^10^
0.54	0.04	0.02		

**Table 8:** Extraction efficiency of 200 nm markers taking into account the results of only the first and fourth extraction steps.

Extraction Efficiency of Markers from Filter *η*			Initial Mass of Markers on the Filter	Initial Number of Markers on the Filter
Average	Standard deviation	Error of the mean	M_o_	n_o_
-	-	-	(mg)	-
0.58			0.202	1.82 × 10^10^
0.60	0.04	0.02	0.213	1.92 × 10^10^
0.52			0.214	1.93 × 10^10^
0.57	0.04	0.02		

## Data Availability

Not applicable.
